# Supplementary material for: Stenotrophomonas maltophilia promotes wheat growth by enhancing nutrient assimilation and rhizosphere microbiota modulation
Source: Front Bioeng Biotechnol. 2025 Apr 17;13:1563670. doi: 10.3389/fbioe.2025.1563670 (PMC12043639; doi:10.3389/fbioe.2025.1563670)

Stenotrophomonas maltophilia promotes wheat growth by enhancing nutrient assimilation and rhizosphere microbiota modulation

Pinki Sharma[1], Rajesh Pandey[2,3*], Nar Singh Chauhan[1*]

[1]Department of Biochemistry, Maharshi Dayanand University, Rohtak, Haryana, India

[2]INtegrative GENomics of HOst-PathogEn (INGEN-HOPE) laboratory, CSIR-Institute of Genomics and Integrative Biology (CSIR-IGIB), Mall Road, Delhi-110007, India.

[3]Academy of Scientific and Innovative Research (AcSIR), Ghaziabad-201002, India.

*Corresponding author
Nar Singh Chauhan (nschauhan@mdurohtak.ac.in)
Rajesh Pandey (rajeshp@igib.res.in)

Running Title: *Stenotrophomonas maltophilia* for sustainable agriculture

Number of Words: 11358
Number of Figures: 11
Number of Tables:02

Supplementary Methods

Supplementary Method SM1 Rhizosphere bacterial culturing and physiological characterization: A 5.0g soil sample was suspended in 20 ml of ultrapure sterile water for physicochemical analysis. The soil suspension was then serially diluted to 10^{-8}, and 0.1 ml of each dilution was spread evenly on a self-devised minimal medium A (pH 7.2), containing urea (200 mg), calcium phosphate (250 mg), ferrous sulfate (20 mg), synthetic sea salt (200 mg), pectin (50 mg), inulin (50 mg), starch (50 mg), sorbitol (50 mg), carboxymethyl cellulose (50 mg), and ammonium sulfate (50 mg) dissolved in 100 ml of distilled water. The culture plates were incubated at 16°C, 25°C, and 37°C to isolate various microbes. Bacterial growth was monitored for 48 hours, and morphologically distinct microbial colonies were sub-cultured at 37°C to obtain pure cultures. Bacterial cultures were screened for antifungal activity using a disc diffusion assay (Balouiri et al., 2016). Rhizosphere microbes from wheat, along with *Rhizoctonia solani* and *Fusarium oxysporum*, were cultured in LB and YEPD broth, respectively, with continuous shaking at 200 rpm for 24 hours at 28°C. A 0.1 ml aliquot of the 1.0 OD600nm overnight fungal culture was evenly spread on PDA plates in sterile conditions. Sterile discs were placed at the center of the plates, and 50 µl of the overnight bacterial culture (A600nm: 1.0) was applied to each disc. The plates were then incubated at 28°C for 48 hours. Fungal growth inhibition was assessed by observing the zone of inhibition around the discs (Balouiri et al., 2016). Growth of BCM_F in anaerobic conditions was checked after its inoculation in a sterile and degassed anaerobic basal broth (Himedia Pvt Ltd). The inoculated tubes were immediately placed in an anaerobic gas jar (Himedia Pvt Ltd), followed by the addition of an anaerobe gas pack (Himedia Pvt Ltd) and an aero indicator tablet (Himedia). The anaerobe gas jar was sealed immediately and placed within an incubator at 37° C for 24 hrs. The absorbance of inoculated media was read at 600nm using a UV visible double-beam spectrophotometer after the incubation.

Supplementary Method SM2 Genomic characterization of microbial isolate BCM_F:

A single purified colony of wheat rhizosphere isolates was inoculated into 5 ml of sterile LB broth (pH 7.0) and incubated at 37°C with continuous shaking at 200 rpm for 24 hours. Following incubation, the culture was centrifuged at 8000 x g for 15 minutes to pellet the microbial cells. The microbial pellet was then used to isolate genomic DNA using the HiPurA™ Bacterial Genomic DNA Purification Kit (Himedia, Cat. No MB 505). The quality of the genomic DNA was assessed via agarose gel electrophoresis, and its concentration was measured using a double-stranded DNA quantification kit (Qubit, Invitrogen, USA).The *Stenotrophomonas maltophilia* BCM_F genome was sequenced on an Illumina MiSeq platform

using the Nextera XT DNA Library Prep kit. The raw sequencing reads were evaluated for quality using FASTQC v0.11.9 (http://www.bioinformatics.babraham.ac.uk/projects/fastqc) and fastQ Validator v0.1.1 (https://github.com/statgen/fastQValidator). Contaminated reads were filtered out to obtain corrected sequences. The genome was assembled de novo using the SPAdes v3.15.1 assembler. To assess the completeness of the predicted gene set in the assembly, we employed the BUSCO v5.0.0 tool with the latest bacterial orthologue catalog (bacteria_odb10). The assembled contigs were then subjected to functional annotation using PROKKA. SSU rRNA gene was extracted, and the BLASTn was performed to identify the taxonomic affiliation of BCM_F.

Supplementary Method SM3 Role of *Stenotrophomonas maltophilia* BCM and BCM_F on seed germination under phytopathogen and saline stress condition

Wheat cultivar -306 seeds were surface sterilized with a 10% sodium hypochlorite solution to remove any microbial contaminants from their surface. Finally, the seeds underwent three to four rinses with sterile distilled water to ensure the complete removal of sterilizing agents. These sterilized seeds were then used for subsequent seed germination experiments.

A seed germination assay was performed by soaking seeds in PBS buffer (pH 7.4) for 16 hours to generate a control dataset. Seeds were incubated with wheat rhizospheric isolates in PBS buffer (pH 7.4) with a microbial density of 10^{11} cells/ml to assess the impact of wheat rhizospheric isolates on seed germination. A total of 10 seeds from each experimental setup were wrapped in germination sheets, inserted in 50 ml culture tubes containing 5 ml Hoagland solution, and incubated for 7 days in the dark at room temperature. The seed germination percentage was calculated by observing the number of seeds germinated after incubation

To assess the role of *Stenotrophomonas maltophilia* BCM and BCM_F on seed germination under saline stress conditions, seeds were soaked in overnight-grown microbial cultures with a cell density of 10^{11} cells/ml, supplemented with varying concentrations of NaCl ranging from 0.0 to 1.0M, and incubated at 37°C for 16 hours, while control seeds soaked directly at different concentrations of NaCl ranging from 0.0 to 1.0M for 16 hours 37ºC. Subsequently, the seeds were wrapped in germination sheets, placed in 50 ml culture tubes containing 5 ml Hoagland solution, and incubated for 7 days in the dark at room temperature. Seed germination percentage, alpha-amylase activity, as well as root and shoot lengths were measured after incubation. All assays were performed in technical triplicates.

To assess the role of *Stenotrophomonas maltophilia* BCM and BCM_F on seed germination under phytopathogen exposure, seeds were soaked in overnight-grown microbial cultures of both wheat rhizosphere microbes and respective fungus with a cell density of 10^{11} cells/ml and

incubated at 37°C for 16 hours, while the seeds treated with fungus only were considered as control. Subsequently, the seeds were wrapped in germination sheets, placed in 50 ml culture tubes containing 5 ml Hoagland solution, and incubated for 7 days in the dark at room temperature. All assays were performed in technical triplicates

Supplementary Method SM3 Wheat rhizosphere microbiota profiling: Wheat cultivar 306 seeds were surface sterilized with a 10% sodium hypochlorite solution to remove microbial contaminants. The seeds were immersed in the solution for 5 to 10 minutes and washed thoroughly with ultrapure water to eliminate any remaining bleach. The surface-sterilized seeds of cultivar 306, were treated with microbial isolates (300 seeds in 20 ml culture (1.0 O.D.)) at seedling stage only and sown in an experimental field at the botanical garden of Maharshi Dayanand University, Rohtak, Haryana, India (28° 52' 44" N, 76° 37' 19" E). Wheat roots were harvested at different Feeks (1.0, 2.0, 3.0, 6.0, 9.0, and 10.5) (https://www.sunflower.k-state.edu/agronomy/wheat/wheatdevelopment.html). Metagenomic DNA from the wheat rhizosphere was extracted using the CTAB method as described by Kumar et al. 2016 (Kumar et al., 2016), followed by purification with the HiPurA soil DNA purification kit (Himedia). The 16S rRNA gene sequences were amplified from the wheat rhizosphere metagenomic DNA using universal primers (27F 5'-AGAGTTTGATCCTGGCTCAG-3'; 1492R 5'-GGTTACCTTGTTACGACTT-3') (Sharma et al., 2024b). Amplified product's quality was assessed using agarose gel electrophoresis. Library preparation was performed using the standard operating protocol provided by nanopore technology (Rapid Sequencing Kit V14 - gDNA (SQK-RAD114) (RSE_9177_v114_revM_16Nov2022) (nanoporetech.com). Sequencing was performed with Flow Cell (R10.4.1) using the single-end midnight pipeline on the Minion MK1C platform (Rapid Sequencing Kit V14 - gDNA (SQK-RAD114) (RSE_9177_v114_revM_16Nov2022) (nanoporetech.com) following default sequencing options (run time 72 hrs, sequence quality >Q8, Sequence length >200bp). The resulting wheat rhizosphere data were analyzed using EPIME 16S rRNA gene pipelines following their default settings (Sharma et al., 2024b).

Supplementary Figure SF1: Microbial abundance at phylum level of taxonomy observed in the rhizosphere of microbial treated and non-treated plants at different Feeks (1.0, 2.0, 3.0, 6.0, 9.0, 10.5). Here 1-316 represents different phylums represented as 1: Proteobacteria, 2: Firmicutes, 3: Actinobacteria, 4: Cyanobacteria, 5: Bacteroidetes, 6: Acidobacteria, 7: Chloroflexi, 8: Synergistetes, 9: Verrucomicrobia, 10: Tenericutes, 11: Planctomycetes, 12: Gemmatimonadetes, 13: Nitrospirae, 14: Deinococcus-Thermus, 15: Thermotogae, 16: Chlorobi

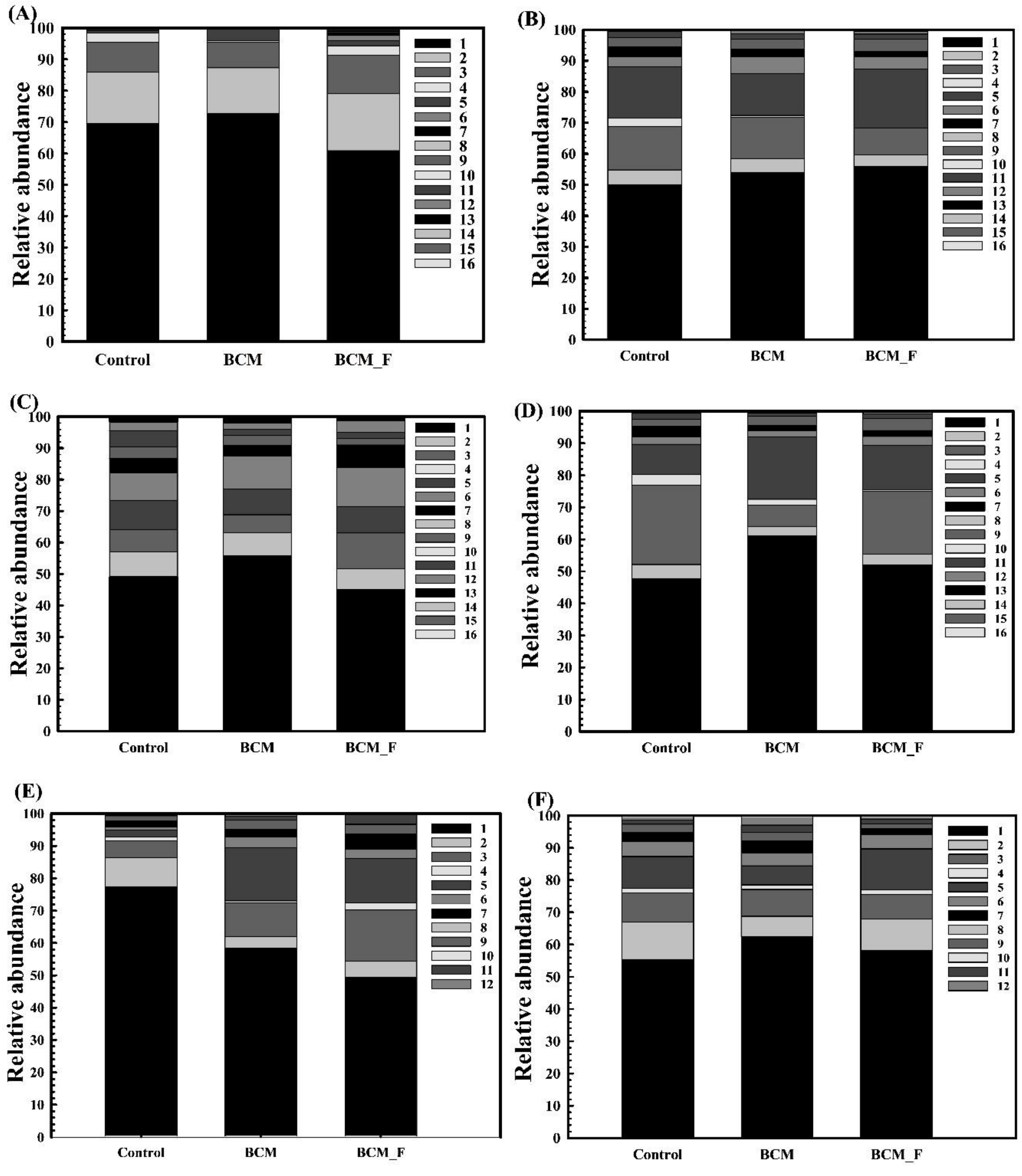

Supplementary Figure SF2: Total sugar content observed at different Feeks (1.0, 2.0, 3.0, 6.0, 9.0, 10.5) in the presence and the absence of microbial inoculants BCM and BCM_F (**A**). Reducing sugar was estimated using DNS assay at different Feeks (1.0, 2.0, 3.0, 6.0, 9.0, 10.5) was assessed in the presence and the absence of microbial inoculants BCM and BCM_F (**B**). The experiment was carried out in triplicates. Plotted values are the mean of triplicate readings and their observed standard deviation.

(A)

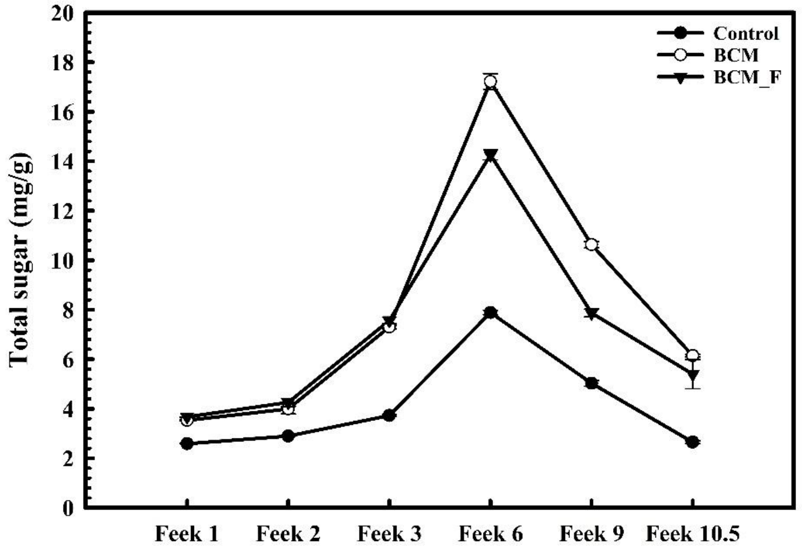

(B)

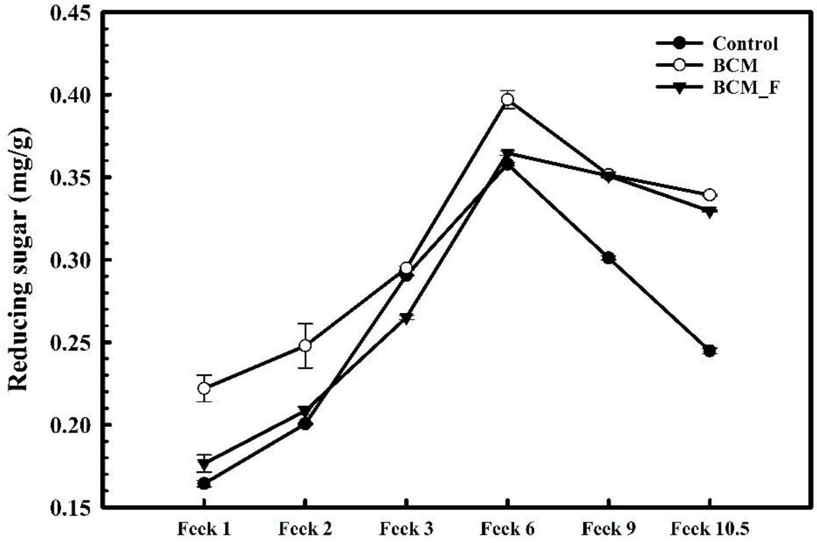

Supplementary Figure SF3: Assessment of nitrate reductase activity at different Feeks (1.0, 2.0, 3.0, 6.0, 9.0, and 10.5) in the presence or absence of microbial groups *Stenotrophomonas maltophilia* BCM and BCM _F (**A**). Assessment of alkaline phosphatase activity at different Feeks (1.0, 2.0, 3.0, 6.0, 9.0, 10.5) in the presence or absence of BCM and BCM_F(**B**). Experiments were carried out in triplicates Plotted values are the mean of triplicate readings and their observed standard deviation.

(A)

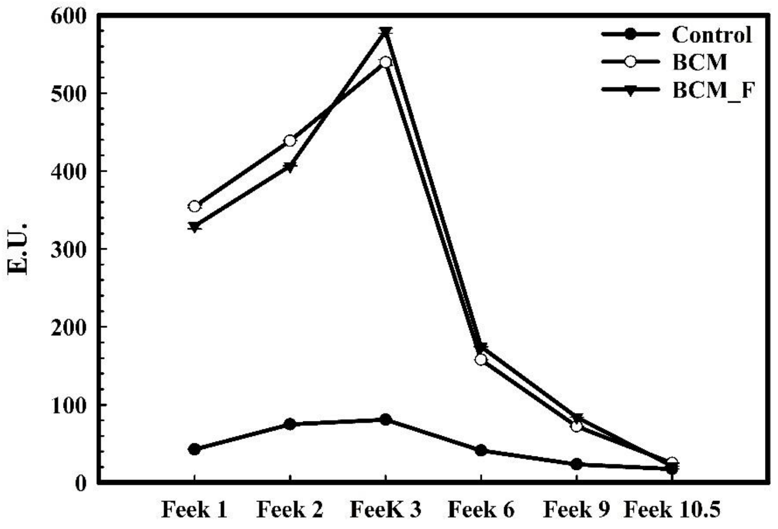

(B)

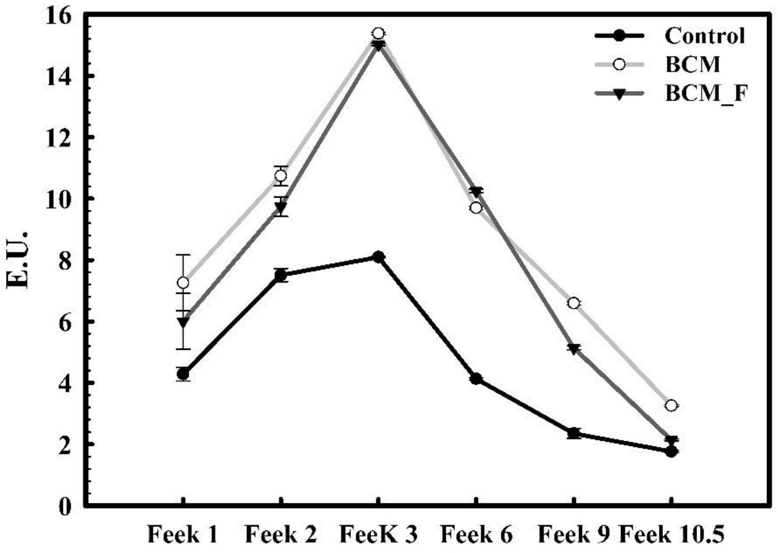

Supplement: Supplementary file 1 [file DataSheet1.pdf]
